# Educating caregivers about fever – what and why?

**DOI:** 10.1038/s41390-024-03134-2

**Published:** 2024-03-14

**Authors:** Damian Roland

**Affiliations:** 1 SAPPHIRE Group, Population Health Sciences, Leicester University, Leicester, UK; 2 Paediatric Emergency Medicine Leicester Academic (PEMLA) Group, Children’s Emergency Department, Leicester Royal Infirmary, Leicester, UK

Fever is a ubiquitous cause of presentation to Primary Care, Emergency Departments and Acute Paediatric services all over the world. Fever is a primary indicator of infection and infection is a leading cause of death and morbidity in children. Why then is there a concept of fever phobia and such concern about the negative effects it has on health seeking behaviour? And why is so much energy used on improving parental education in how to manage the child with a fever?

There are two related underlying principles here; the first that fever in children is common but the negative consequences of fever are rare. Numbers vary depending either on economic status of the country, access to healthcare services and incidence of serious illness but infants may have an annual incidence of 5–7 episodes of fever. General Practitioners or Family Doctors being consulted around 3.7 times per year specifically about fever.^1^ Fever episodes will largely be secondary to viral illnesses which are self-limiting in nature and have no long term consequence for the child. In fact there is evidence fever is a positive attribute and mortality, in admitted adult patients, is increased by regular prescription of antipyretics to reduce it.^2^

The second is concern about fever is disproportionate to its outcome. Schmitt^3^ in 1980 highlighted the extreme reaction to fever some parents have. 20 years on work by Crosetti^4^ demonstrated little had changed. (Table 1). There hasn’t been a formal replication of this work in the 2020 s but there is no reason to believe, given that fever remains the most common reason that children attend Emergency Departments, that this has changed.

**Table 1:** Parental reported harmful effects of fever.

Outcome of Concern	Schmitt (*n* = 81) 1980 [3]	Crocetti (*n* = 341) 2001 [4]
Brain Damage	45%	21%
Seizure	15%	32%
Delirium	12%	1%
Death	8%	14%
No response	6%	9%
Coma	4%	2%
Dehydration	4%	4%
Blindness	3%	1%
Really Sick	1%	2%
Other		14%

Lynch et al.’s recent publication^5^ in Paediatric Research also evidenced the need for continued education. Their very practical (and importantly reproducible) education programme demonstrated only 41% of caregivers attending a Children’s Emergency Department could define what constituted a significant temperature (38 °C or 100.4^0^F) in a child correctly. Their intervention of written and video material appeared successful with correct answers in 94% of the 48 caregivers in the post-intervention group. It also improved a number of secondary objectives such as myth-busting the need for tepid sponging and that antipyretics are always needed regardless of the child’s level of distress.

Those reading this paper may be struck by the low number of caregivers appreciating what defines a fever in a medical context. However this may well be a little paternalistic. What constitutes a fever is subject to debate. In fact, even in the United Kingdom’s National Institute for Health and Social Care excellence (NICE) guidance on management of the Feverish Child, a specific temperature is only mentioned in the context of a risk assessment for a less than six month old infant. Why would parents and carers know what the appropriate cut off is to be concerned about? This knowledge doesn’t form part of a curriculum during primary or secondary school education and is probably only given to parents by midwives and health visitors at the birth of the child, a time period when information is easily forgotten. This latter fact is relevant to Lynch et al.’s study as they acknowledge the subsequent recall of this information was not measured. How long a parent or carer may retain a given number is not clear. Also, while to the study teams’ credit they produced information in both written and audiovisual formats (an important factor in safety netting^6^), how families would access this information again and which resources they would turn to isn’t clear. Access to information in the future depends on not losing the leaflet you have been given or, if via the internet, the website address not changing. Access is also dependant on not being subject to digital poverty or that the language the health information is written in is your first language.

One question that also hasn’t been answered is how much fever education drives fever phobia itself. An adage in paediatrics is “*treat the child, not the fever*”. Do families, apart from those in high risk groups such as neonates and the immunosuppressed, even need to own a thermometer at all? Does the ‘act’ of educating about a temperature boundary just re-inforce that there is such a boundary to be concerned about? Ultimately is the positive information (i.e. not needing to tepid sponge and the risk of serious illness being very low) negated by the fact that we as paediatricians still require to know when a child definitely has a fever or not? If we think it’s important, why shouldn’t the caregivers? And so when they do have a confirmed fever they are going to worry about it; regardless of how much reassurance they have been given that it’s probably not that important in the well appearing child. Lynch et al. have shown how it is possible to rapidly improve caregiver knowledge. The paediatric community now have to determine what that knowledge should be.
